# Supplementary material for: The policy effect of green finance reform and innovations: Empirical evidence at the firm level
Source: PLoS One. 2022 Dec 1;17(12):e0278128. doi: 10.1371/journal.pone.0278128 (PMC9714720; doi:10.1371/journal.pone.0278128)
Supplement: S1 Appendix — (DOCX) [file pone.0278128.s001.docx]

**Appendix A: List of covered pilot regions**

Shenzhen, Zhuhai, Guangzhou, Ningbo, Foshan, Guiyang, Nanchang, Hangzhou, Urumqi, Dongwan, Jiangmen, Guiyang, Shaoguan, Jiangmen, Chaozhou, Zhaoqing, Maoming, Yichun, Zhuhai, Zhongshan, Meizhou, Shaoxing, Shangrao, Shantou, Ganzhou, Nanchang, Taizhou, Nanchang, Lishui, Xieyang, Huzhou, Ningbo, Jinhua, Quzhou, Wenzhou, Jiaxing, Jingdezhen, Lishui, Meizhou, Jinhua, Karamay, Anshun, Qingyuan, Zunyi, Yingtan, Pingxiang, Zhanjiang, Xinyu
